# Biodiversity of Mosquitoes (Diptera: Culicidae) with Emphasis on Potential Arbovirus Vectors in East Azerbaijan Province, Northwestern Iran

**Published:** 2019-03-30

**Authors:** Azim Paksa, Mohammad Mahdi Sedaghat, Hassan Vatandoost, Mohammad Reza Yaghoobi-Ershadi, Seyed Hassan Moosa-Kazemi, Teimour Hazratian, Alireza Sanei-Dehkordi, Mohammad Ali Oshaghi

**Affiliations:** 1 Department of Medical Entomology and Vector Control, School of Public Health, Tehran University of Medical Sciences, Tehran, Iran; 2 Institute for Environmental Research, Tehran University of Medical Sciences, Tehran, Iran; 3 Departmemt of Parasitology, Faculty of Medicine, Tabriz University of Medical Sciences, Tabriz, Iran; 4 Department of Medical Entomology and Vector Control, Faculty of Health, Hormozgan University of Medical Sciences, Bandar Abbas, Iran; 5 Infectious and Tropical Diseases Research Center, Hormozgan Health Institute, Hormozgan University of Medical Sciences, Bandar Abbas, Iran

**Keywords:** Diversity, Ecology, Mosquitoes, Arbovirus vectors, Iran

## Abstract

**Background:**

The abundance, diversity, distribution and ecology of mosquitoes (Diptera: Culicidae), especially arbovirus vectors are important indices for arthropod-borne diseases control.

**Methods:**

Larvae and adult mosquitoes were collected using the standard methods from different habitats in nine localities of three counties in the East Azerbaijan Province, Northwestern Iran during June to October 2017. In addition, species richness (R), Simpson’s diversity index (D), Shannon–Wiener index (H′) and evenness (E) as measures of diversity, were calculated.

**Results:**

Overall, 1401 mosquito specimens including 1015 adults and 386 larvae were collected in the study area. The properties of geographical larval habitats were recorded. Four genera along with 10 species were collected and identified, including *Anopheles hyrcanus*, *An. maculipennis* s.l., *An. superpictus* s.l., *Aedes caspius*, *Ae. vexans*, *Culex pipiens*, *Cx. theileri*, *Cx. perexiguus*, *Culiseta longiareolata* and *Cs. subochrea.* Among the three counties, Ahar region presented the highest species richness (R: 1.5) and diversity values (D: 0.79, H′: 1.74, E: 0.73).

**Conclusion:**

This study provides important information on the diversity, distribution and ecology of ten mosquito species in the region. This information leads to a better understanding of mosquito population dynamics in relation to vector control measures.

## Introduction

Mosquitoes (Diptera: Culicidae) are sub-order of Nematocera and Culicidae family, which are the medically-important species for malaria transmission, types of filariasis, types of encephalitis and other arboviral diseases (1–2). The Culicidae family includes 2 subfamilies, 11 tribes; The Culicidae family includes 2 subfamilies, 41 Families, 11 tribes, 113 genera and 3563 species (1–4). The most important genera of the Culicidae family are *Anopheles*, *Culex* and *Aedes*. Among the Culicidae, the *Aedes* species have the most various species and are extremely important in medical cases (5).

Mosquitoes play a crucial role in transmission of some important diseases such as malaria, filariasis, dengue fever, yellow fever, chikungunya, West Nile virus and Zika virus which are today among the greatest health problems in the world (6–9).

Mosquitoes larvae have the power to colonize and live in a wide variety of natural and artificial habitats, such as temporary and permanent water resources, unclean and clean water, large or small water resources, stagnant or stream waters and even the smallest places where water is gathered, such as buckets of water, pots, tires, bromeliads, animal feet prints and plant leaf axes (10–11). Adult mosquitoes are very diverse bionomically, for example, they are too diverse in host searching, biting behaviors, dispersal and reproductive strategies (9). Two factors (abiotic and biotic) affect mosquito life cycle. The biotic factors contain adult sugar and blood meal types, species communications, interactions and natural enemies. Physicochemical attributes of larval habitats are abiotic factors, which include the type and contents of water, temperature and rainfall. There are complex interactions between these factors that significantly affect mosquito ecological adaptability and vectorial capacity for disease transmission with significant concepts for vector management and control at the local and regional levels (12–13). Accordingly, investigating biotic and abiotic factors for various mosquito fauna make it easier to monitor potential modifications of larval habitats affected by rains, global climate change and man-made activities (12–13).

*Aedes caspius* likely transmit the human pathogen, such as *Spiroplasma sabaudiensc* and *Crisiulospora aedis* (14). These mosquitoes transmit Rift Valley fever virus (RVF), West Nile virus (WNV) and Tahyan virus (15). In the region of Palearctic, *Ae. caspius* inhabits in lakes, pools, shores of Great Britain, and fresh-water and lower salt marshes in the continental parts of Europe, Russia, Mongolia, northern China, Pakistan, northeastern Africa, Asia Minor, and the Persian Gulf (17–18).

*Aedes vexans* has several subspecies, the typical species is *Ae. vexans vexans*, and the subspecies *Ae. nipponii vexans* in East Asia (19) and *Ae. vexans arabiensis* in Saudi Arabia, Yemen and Africa (3). This species is extremely important in medicine and has more than 30 different viral diseases, including Eastern horse encephalitis, Japanese encephalitis (20), California encephalitis, Western horse encephalitis (21), as well as other pathogens such as Tularemia and *Dirofilaria immitis* (22–23). *Aedes vexans* along with *Ae. aegypti* and *Cx. quinquefasciatus*, have the highest global distribution among other mosquitoes in the world (24–25).

Several studies were conducted on composition, distribution and ecology of mosquitoes in Iran (26–44). Considering the fact that in the East Azarbaijan Province, some vector mosquitoes of diseases such as West Nile, Dirofilariasis and malaria present, study on the composition, distribution and ecology of mosquito species is very important in different aspects of vector control programs. This study will add important information about the composition, distribution, ecology and diversity of mosquito species such as establishment of mosquitoes in this region. This new and important information will help us to correctly control and monitor strategies of disease vectors, such as *Ae. caspius*, *Ae*. *vexans*, *Cx. pipiens* and *Cx. theileri*. This information helps us to prevent important vectors from increasing and establishment in this area due to change in human activities and weather; thus, the risk of transmission of disease by mosquitoes gets minimized.

The aim of this study was to determine composition, distribution and some ecological aspects of mosquitoes in East Azerbaijan Province, Iran which is high medically importance from the point of view of the arboviral vectors.

## Materials and Methods

### Study area

**Fig. 1.**
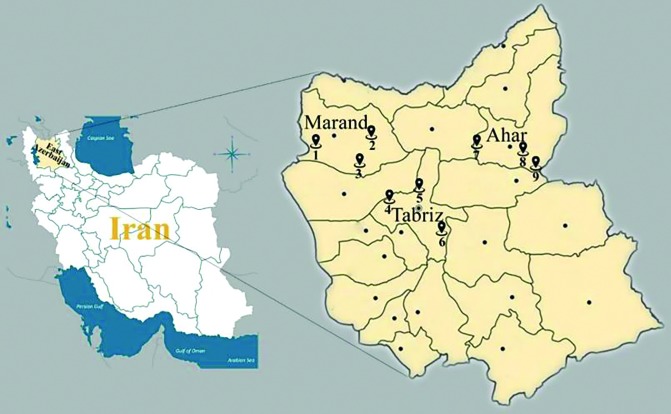
Map of Iran and locations of study areas in East Azerbaijan Province, Iran

East Azerbaijan Province is located in northwestern of Iran between 39° 26′–36° 45′ N latitudes and 45° 5′–48° 22′ E longitudes. The province covers an area of approximately 47,830km^2^, it has a population of around four million people (Fig. 1, Table 1).

**Table 1.** Geographical characters of the locations were studied

**No.**	**Locations**	**Latitude (N)**	**Longitude (E)**	**Altitude (M)**
**1**	Ghareh Tappeh	38° 26′ 9.758″	45° 35′ 7.828″	1296
**2**	Dolat Abad	38° 45′ 53.988″	45° 49′ 19.418″	1297
**3**	Zinab	38° 9′ 9.654″	45° 52′ 53.218″	1302
**4**	Khaje dizaj	38° 12′ 21.030″	46° 16′ 39.659″	1325
**5**	Hojjaj Park	38° 11′ 16.210″	46° 23′ 45.659″	1336
**6**	Chavan	37° 59′ 9.952″	46° 23′ 32.164″	1759
**7**	Sattar Khan Dam	38° 46′ 15.552″	46° 8′ 22.763″	1429
**8**	Razin	34° 24′ 53.932″	47° 8′ 24.629″	1435
**9**	Yavarkandi	38° 38′ 45.622″	47° 21′ 97.143″	1706

This province contains 19 counties. Tabriz City is center of the province which 1360m above sea level. The mean annual rainfall is about 300mm. The average relative humidity changes are from 44%. The averages of the maximum and minimum temperatures are 17.7 °C and 6.8 °C, respectively, and the average temperature is 12.3 °C. The province includes arid and semiarid climates (Fig. 1, Table 1).

### Mosquito collection, site selection and species identification

For mosquito collection, nine sites with different biotopes in three counties of Ahar, Marand, and Tabriz from East Azerbaijan Province were selected.

Collection of mosquito’s larvae was performed in different habitats using the standard dipping technique (using 350ml Clark’s dippers) and whirl pack bags (45) (Table 2). Light trap, mosquito net and aspirator was used for adult mosquito collection. Collection of mosquitoes was carried out during June to October 2017. Larvae and adult mosquitoes were identified by morphological characters (46).

**Table 2.** Details of mosquitoes collected from larval and adult habitats in East Azerbaijan Province during June–October 2017

**County**	**Location**	**species**	**Number**		**Total**	**Percent**

			**Larvae**	**Adult**		
**Ahar**		*Cs. longiareolata*	47	0	47	23
	Yavarkandi	*Cx. pipiens*	28	4	28	14
		*Cx. theileri*	0	2	2	1
		*Ae. caspius*	7	0	7	3.5
		*Ae. vexans*	3	6	9	4.5
	Razin	*Cx. pipiens*	6	10	16	8
		*Cx. theileri*	15	7	22	11
		*Cx. perexiguus*	13	0	13	6.5
		*An. superpictus* s.l.	4	0	4	2
		*Cs. subochrea*	12	0	12	6
	Sattar Khan Dam	*Cx. theileri*	34	0	34	17
		*Cx. pipiens*	4	3	7	3.5

	**Subtotal**		173	32	205	100

**Tabriz**		*Ae. caspius*	16	43	59	7.76
		*Cs. longiareolata*	13	1	14	1.85
	Hojjaj park	*Cx. pipiens*	10	17	27	3.55
		*Cx. theileri*	15	12	27	3.55
		*Cx. perexiguus*	7	0	7	.93
		*Cs. subochrea*	0	1	1	.13
		*Cx. pipiens*	23	73	96	12.63
	Khage dizaj	*Cx. theileri*	417	39	456	60
		*Ae. vexans*	0	1	1	.13
		*Cx. pipiens*	11	13	24	3.16
	Chavan	*Cs. longiareolata*	48	0	48	6.31

	**Subtotal**		560	200	760	100

**Marand**		*Cs. longiareolata*	186	83	269	61.6
		*Cx. pipiens*	3	2	5	1.4
		*Ae. vexans*	2	13	15	3.3
	Ghareh Tappeh	*An. maculipennis* s.l.	2	2	4	.9
		*An. hyrcanus*	1	0	1	.2
		*Cx. theileri*	0	17	17	3.9
	Dolat Abad	*Cx. theileri*	34	9	43	9.8
		*Cx. pipiens*	6	14	20	4.6
		*Cs. longiareolata*	32	0	32	7.3
		*Cx. pipiens*	7	8	15	3.3
	Zinab	*Cx. theileri*	0	6	6	1.4
		*An. maculipennis* s.l.	5	0	5	1.4
		*An. hyrcanus*	4	0	4	.9

**Subtotal**			282	154	436	100

**Total**			1015	386	1401	100

### Physical and biological characteristics of larval habitats

In these investigation characteristics of mosquitoes larval habitats was studied. The ecological characteristics containing geographical properties of collecting localities (latitude, longitude and altitude), type of habitat (stagnant, stream, seepage and water container), vegetation situation (presence or lack of vegetation), kind of vegetation (Null, Poaceae, Typhaceae, Dispacea and Acorus species), water situation (turbid and clear), exposure to sunlight (full, partial sunlight and covered or shaded), depth, substrate type (muddy, sandy, rocky and concrete), distance from animal and human houses and physicochemical attributes such as water temperature and pH were recorded visually or using special equipment (9, 47).

### Data analysis

The species richness (R: Margalef index), unified indices (D: Simpson’s diversity index and H′ Shannon–Wiener index) and evenness (E: distribution of abundances among the species) as measures of a diversity, were calculated for East Azerbaijan Province and different study counties.

The formulae and their rationale in the present study are summarized below:

$$\begin{array}{l} R=\frac{S-1}{\ln\;\;N}\\ D=1-\sum_{i=1}^{s}\frac{n_{i}(n_{i}-1)}{N(N-1)}\\ H^{\prime }=\sum_{i=1}^{s}\left(p_{i})[\ln(p_{i})\right]\\ E=\frac{H^{\prime }}{\ln\;(s)} \end{array}$$

## Results

Totally, 1401 mosquitoes were collected from nine sites in East Azerbaijan Province during June–October 2017, including 1015 adult and 386 larvae. The properties of geographical larval habitats (Latitude, Longitude and altitude) have been shown in (Fig. 1, Table 1). Four genera along with ten species were collected and identified, including *An. hyrcanus*, *An. maculipennis* s.l., *An. superpictus* s.l., *Ae. caspius*, *Ae. vexans*, *Cx. pipiens*, *Cx. theileri*, *Cx. perexiguus*, *Cs. longiareolata* and *Cs. subochrea* (Table 2).

To our knowledge based on literature, for the first time four species of, *Ae. caspius*, *Ae. vexans*, *Cx. perexiguus*, and *Cs. subochrea* species from Ahar and two species of *Ae. vexans* and *Cx. perexiguus* were reported from Tabriz County. Considering that no investigations have been already carried out in Marand, all the collected species comprising *An. hyrcanus*, *An. maculipennis* s.l., *Ae. vexans*, *Cx. pipiens* and *Cs. longiareolata* (Table 2) are new report for the region.

*Anopheles maculipennis* s.l., *Ae. caspius*, *Ae. vexans*, *Cx. pipiens*, *Cx. theileri*, *Cs. longiareolata* and *Cs. subochrea* species were collected at both adult and larval stages but *An. hyrcanus*, *An. superpictus* s.l., and *Cx. perexiguus* species were collected only at the larval stages (Table 2).

In adult stage, *Cx. pipiens* (37.3%), *Cx. theileri* (23.8%), *Cs. longiareolata* (21.8%), *Ae. caspius* (11.14%), *Ae. vexans* (5.18%) were the most abundance species respectively, However, *An. maculipennis* s.l. (0.52%) and *Cs. subochrea* (0.26%) were the least abundance species (Table 2).

At the larval stage *Cx. theileri* (50.72%), *Cs. longiareolata* (32.12%) and *Cx. pipiens* (9.75%) were the most prevalent species respectively but in contrast *Ae. caspius* (2.27%), *Cx. perexiguus* (1.97%), *Cs. subochrea* (1.09 %), *An. maculipennis* s.l., (0.69%), *An. hyrcanus* (0.5%) and *An. superpictus* s.l. (0.39%), were the least abundance species respectively (Table 2).

Species such as *Cx. pipiens*, *Cx. theileri*, *Cs. longiareolata* and *Ae. vexans* have a wide distribution in the study areas but some species such as *An. hyrcanus*, *An. maculipennis* s.l., *An. Superpictus* s.l., and *Cs. subochrea* have been collected from limited areas (Table 2).

### Characteristics of mosquito larval habitats of mosquitoes

In Ahar County located in northeastern East Azerbaijan Province, there were three larval sites such as Yavarkandi, Razin and Sattar Khan Dam (Table 3, Fig. 1). Sattar Khan Dam and Razin larval sites had seepage and stagnant water respectively. Both of the larval sites had turbid water with muddy substrate and shallow depth. In addition, these sites were covered from sunlight with Null, Poaceae, Typhaceae and Dispacea vegetation. Those were more than two kilometers away from human and animal houses. The Sattar Khan Dam larval habitat had 200 meters distance from animal space and its field temperature was 13 °C. In contrast, Razin larval site had 500 meters distance from animal space and its field temperature was 9 °C. In these two larval habitats harbored *Ae. caspius*, *Ae. vexans*, *Cx. theileri*, *Cx. pipiens*, *Cx. perexiguus*, *Cs. subochrea* and *An. superpictus* s.l. (Table 2). Yavarkandi larval site had stagnant and clear water without vegetation, with muddy substrate and exposed to sunlight. It was more than two km away from human and animal houses. In the Yavarkandi larval site the field temperature was 23 °C. In this larval habitat, two species of *Cs. longiareolata* and *Cx. pipiens* were collected (Table 3).

**Table 3.** Characteristics of mosquito larval collection sites in Ahar, Tabriz and Marand counties of East Azerbaijan Province during June–October 2017.

**County**	**Location**	**Type**	**Turbid**	**Exposed**	**Vegetation**	**DHH**	**DAH**	**Depth**	**Sub.**	**T**
**Ahar**	Yavarkandi	Stagnant	Clear	Exposed	Without	>2km	>2km	<1m	Muddy	23
	Razin	Stagnant	Turbid	Covered	Null, Poaceae, Typhaceae, Dispacea	>2km	500m	Shallow	Muddy	9
	Sattar Khan Dam	Seepage water	Turbid	Covered	Null, Dispacea	>2km	200m	Shallow	Muddy	13

**Tabriz**	Hojjaj park	Stagnant	Turbid	Partial	Poaceae	20m	>2km	Shallow	Muddy	17
	Khage dizaj	Stagnant	Turbid	Covered	Null, Acorus species	1km	100m	<1m	Muddy	16
	Chavan	Water container	Clear	Exposed	Without	2m	10m	1m	Concrete	27

**Marand**	Ghareh Tappeh	Stagnant	Clear	Exposed	Without	ND	ND	1m	Sandy	18
	Dolat Abad	Stagnant	Clear	Partial	Null, Dispacea	>2km	>2km	Shallow	Muddy	19
	Zinab	Stream	Clear	Exposed	Without	100m	100m	Shallow	Rocky	17

ND: not determined, Tur: Turbidity, Exp: Sun exposure, Veg: Vegetation type, DHH: Distance from the nearest human houses, DAH: Distance from the nearest animal house, Sub: Substrate type, T: Temperature.

Tabriz larval site is located in the central East Azerbaijan Province and contains three regions of Hojjaj Park, Khaje-dizaj and Chavan (Table 3, Fig. 1). Both regions of Hojjaj Park and Khaje-dizaj had stagnant and turbid water with muddy substrate. The Hojjaj Park had the following conditions: the parts of larval habitat were covered with *Poaceae* plants and its water depth was shallow, also it had more than two kilometers distance from animal and human houses. The field temperature was 17 °C in this site (Table 3). This site contain**s** *Ae. caspius*, *Cx. theileri*, *Cx. pipiens*, *Cx. pere*xiguus and *Cs. longiareolata* (Table 2). The Khaje-dizaj site was covered with Null and Ziziphu plants. Its depth was less than one meter and its field temperature was 16 °C. It had one-kilometer distance from human houses and about 100 meters from animal sites (Table 3). In this larval habitat, *Cx. theileri* and *Cx. pipiens* were collected (Table 2). Chavan larval habitat was a water container with substrate cement and clear water that had no plants and was exposed to sunlight. It had a very short distance with human and animal houses (About 2–10m), which had one meter depth and 27 °C (Table 3). In this larval habitat *Cs. longiareolata* and *Cx. pipiens* were collected (Table 2).

In Marand with three larval sites of Ghareh Tappeh, Dolat Abad and Zinab located in northwest East Azerbaijan Province (Fig. 1). Ghareh Tappeh and Dolat Abad habitats had stagnant and clear water. Ghareh Tappeh site had no plants with sandy substrate and shallow depth that was exposed to sunlight. It had one meter depth and 18 °C field temperature (Table 3). *Culiseta longiareolata*, *Cx. pipiens*, *Ae. vexans*, *An. maculipennis* s.l. and *An. hyrcanus* were collected in this larval habitat (Table 2). Dolat Abad site was shallow with muddy substrate and 18 °C field temperature. The parts of larval habitat were covered with Null and Dispacea plants and it had more than two kilometers distance from human and animal houses (Table 3). In this larval habitat *Cx. theileri* and *Cx. pipiens* were collected (Table 2). Zinab site was stream and clear water with rocky substrate and shallow depth. It had no plants and exposed to sunlight with 17 °C temperature. It had about 100 meters distance from human and animal houses (Table 3). In this larval habitat, specimens of *Cx. pipiens*, *Cs. longiareolata*, *An. maculipennis* s.l. and *An. hyrcanus* were collected (Table 2, Fig. 1).

According to Table 2 and 3, larvae of mosquitoes occupied all different types of the habitats. In addition, our results showed that *Cx. pipiens*, *Cs. longiareolata* and *Cx. theileri*, respectively, had the most distribution and adaptation to different types of larval habitats. These three species were collected in most larval habitats but in contrast, *Cx. perexiguus* was found only in the shallow water that was stagnant, turbid, covered with plants and with muddy substrate. In addition, distribution of *Cs. subochrea* was limited and only was collected in the seepage, stagnant and turbid water habitats with muddy substrate and shallow depth. *Anopheles* was found only in stream, stagnant and clean water with muddy substrate that was exposed to sunlight. *Aedes vexans* and *Ae. caspius* were found mostly in larval habitats with turbid water and they were covered with different types of plants. Only two *Ae. vexans* specimens were found in clean water that was exposed to sunlight with muddy substrate (Table 2, 3). In our study, the temperature and pH ranges were 9 °C to 27 °C and 7–8 respectively.

There were differences in the species diversity, as indicated by the values of Simpson’s diversity index, Shannon-Wiener index (H’), and evenness and species richness of the mosquito fauna among the study areas of East Azerbaijan Province (Table 4). The species richness and the three indices were found to be maximal in Ahar County (R: 1.5, D: 0.79, H’: 1.74, E: 0.73), whereas the estimated diversity (D: 0.49, H’: 1.01), and richness (R: 0.82) were the lowest in Marand county.

**Table 4.** The species richness (R), Simpson’s diversity index (D), Shannon-Weiner diversity index (H′), and evenness (E) of the collected mosquito species in East Azerbaijan Province during June–October 2017

**Loc.**	**No.**	**S**	**R**	**D**	**H′**	**E**
**Marand**	436	6	0.823	0.491	1.008	0.563
**Tabriz**	760	7	0.905	0.547	1.069	0.550
**Ahar**	205	9	1.503	0.789	1.735	0.727
**East Azerbaijan Province**	1401	11	1.380	0.694	1.513	0.631

## Discussion

This research is the first study on distribution, diversity and ecology of mosquitoes, with emphasis on *Ae. capius* and *A. vexans* as potential arbovirus vectors in East Azerbaijan Province, northwestern of Iran. The East Azerbaijan Province contains diverse geographical areas with different weather conditions. These diverse conditions can provide suitable environment for the establishment of different species of mosquitoes and justify the variety of mosquito species in this region. This study showed many mosquito species had ecological adaptations. In spite of these ecobiological characteristics, the ecology of mosquitoes present in East Azerbaijan Province is largely unknown. In this investigation we tried to study distribution and ecology of mosquitoes in three northeastern, northwest and central regions of East Azerbaijan Province, where various mosquito vectors of malaria and arboviruses are present (48–49). Some studies had been conducted on fauna and checklist of mosquitoes in parts of this region (48), although to our knowledge no studies have been done on the ecology of mosquitoes in these regions.

In current study, 4 genera and 10 species were collected and identified. For the first time, *Ae. caspius*, *Ae. vexans*, *Cx. perexiguus*, *Cs. subochrea* species were reported from Ahar county. Also for the first time, *Ae. vexans* and *Cx. perexiguus* species were reported from Tabriz county. As well as for the first time, *An. hyrcanus*, *An. maculipennis* s.l., *Ae. vexans*, *Cx. pipiens*, *Cx. theileri* and *Cs. longiareolata* species were reported from Marand county. Some previous studies had found only *An. hyrcanus* in northwestern Iran (49–50) and other study identified *An. claviger*, *An. hyrcanus*, *An. maculipennis* s.l., *An. pseudopictus*, *An. sacharovi* and *An. Superpictus* s.l. in East Azerbaijan Province. In the present study, we reported *An. hyrcanus*, *An. maculipennis* s.l. and *An. Superpictus* s.l.. In previous studies, *Cx. pipiens* and *Cx. theileri* were reported in these regions (48), but *Cx. perexiguus* was not found in these regions. In this study, three species of *Culex* genus were reported such as *Cx. pipiens*, *Cx. theileri* and *Cx. perexiguus* that *Cx. pipiens* and *Cs. longiareolata* species were the most abundant. *Aedes caspius* and *Ae. vexans* were found only from the Kaleybar region (49), In our study, for the first time, *Ae. caspius* and *Ae. vexans* were found in Ahar, Marand and Tabriz, where previous study did not report these species in these areas (48). One study reported *Cs. annulata* and *Cs. subochrea* in West Azerbaijan Province and *Cs. subochrea* in East Azerbaijan Province (51). *Culiseta longiareolata* species was reported as the most abundant in Kermanshah, Kurdistan and Sistan and Baluchistan provinces (52). In this study, two members of *Culiseta* genus were found, including *Cs. longiareolata* and *Cs. subochrea* where *Cs. longiareolata* species was the most abundant species.

Comparing the results of our study with a recent study in West Azerbaijan Province (47) showed that six species *An. maculipennis* s.l., *An. superpictus* s.l., *Cx. pipiens*, *Cx. theileri*, *Cs. longiareolata* and *Ae. caspius* were common between West and East Azerbaijan provinces. The results of our study compared with the results of research conducted in Zanjan Province (53), showed that five species (*An. maculipennis* s.l., *An. superpictus* s.l., *Cx. pipiens*, *Cx. theileri*, and *Cs. longiareolata*) were common between these two provinces. The comparison the results of our study with a recent study conducted in Kurdistan Province (54) showed that six species (*An. maculipennis* s.l., *An. superpictus* s.l., *Cx. theileri*, *Cx. pipiens*, *Cs. longiareolata* and *Ae. caspius*) were common between in provinces. In Turkey (55), and in the provinces of Ardebil, Kurdistan and West Azarbaijan, *Cx. theileri* and *Cx. pipiens* were dominant and most abundant species (56) and our study results confirm this information. Our results showed that *Cx. theileri* (50.72%), *Cs. longiareolata* (32.12%) and *Cx. pipiens* (9.75%) were dominant and the most abundant species in this province.

The climate changes and biotic and abiotic environment factors including plants, temperature and rainfall ranges, significantly affect the type and frequency of larval mosquito habitats. These factors, in turn, affect the number of mosquito species, larval stages, longevity, behavior and adult development. As a result, the transmission of diseases through mosquitoes is directly affected by environmental factors (57–58).

The presence of plants as a source of sugar for mosquitoes is very important, that influence both larval and adult stage development (59–70). Plants provide energy for mosquitoes, As a result, survival rate increased and longevity of the mosquito longer than the extrinsic incubation period of parasite, therefore the incidence of disease increases (61–62). In our study, five types of plants such as Null, Poaceae, Typhaceae, *Carex dipsacea* and *Acorus* species were found in relation to mosquito larval habitats. To our knowledge, there is no study on plant species associated with mosquito species in Iran. In our study, various species of mosquitoes such as *Anopheles*, *Culex*, *Aedes* and *Culisita* were found in relation with various plants, therefore this study confirms the results of previous research. *Aedes vexans* and *Ae. caspius* have not been reported earlier in these areas. These results are likely to show the effects of climate changes and human activities on the distribution of these species in these regions.

Physicochemical properties of larval habitats regulate the abundance of mosquito species, for example, *Anopheles* species were found more in natural larval habitats and *Culex* species in artificial larval habitats (63). In our study, *Anopheles* species were found in natural habitats, while *Culex* species were collected from different types of larval habitats, indicating these mosquitoes can live in a wide range of water habitats. Larval habitats in this study were stagnant, stream and seepage, water container, turbid and clear water, sun exposed or covered from sunlight–rocky or muddy substrate and shallow depth. Studies showed that some of *Culex* larval species were found alone or along with other mosquitoes, such as *Anophele* and *Aedes* (64–67) which has been observed in our study.

There was a significant relationship between the distance of larval habitats of *Anopheles* mosquitoes, human and animal sites, besides, *Anopheles* mosquitoes are found more often near human and animal houses (68). Our study showed that there was a significant correlation between the distances of larval habitats of *Anopheles*, *Ae. caspius*, *Ae. vexans* and *Cx. theileri* mosquitoes with human and animal sites, because the larval habitats of these mosquitoes were found more near to human and animal houses. However, this correlation was not observed between the larval habitats of *Cx. pipiens* and *Cs. longiareolata* with human and animal houses, because the habitats of these mosquitoes were found at various intervals from human and animal houses.

## Conclusion

Arbovirus vectors such as *Ae. caspius* and *Ae. vexans* along with *Cx. pipiens* and *Cx. theileri* are well adapted to a broad range of habitats and climatic conditions. Determining of distribution and full description of ecology of arboviral vectors under local eco-demographic conditions in the East Azerbaijan Province have provided important ecological information on establishment of important mosquito borne diseases. This new information will help us to correctly control and monitor strategies of disease vectors, and in this way prevent important vectors such as ae. *Ae. Caspius*, *Ae. vexans*, *Cx. pipiens* and *Cx. theileri* to be increased and established in this area due to changing human activities and weather changes. These strategies help minimizing the risk of transmission of disease by mosquitoes.
